# Aspects of bovine herpesvirus-1 infection in dairy and beef herds in the Republic of Ireland

**DOI:** 10.1186/1751-0147-53-40

**Published:** 2011-06-23

**Authors:** DJ Bosco Cowley, Tracy A Clegg, Michael L Doherty, Simon J More

**Affiliations:** 1 MSD Animal Health, 4th Floor, Junction House, Airton Road, Tallaght, Dublin 24, Ireland; 2 School of Agriculture, Food Science and Veterinary Medicine, University College Dublin, Belfield, Dublin 4, Ireland; 3 Centre of Veterinary Epidemiology and Risk Analysis, School of Agriculture, Food Science and Veterinary Medicine, University College Dublin, Belfield, Dublin 4, Ireland

## Abstract

**Background:**

Infection with bovine herpesvirus-1 (BHV-1) causes a wide range of disease manifestations, including respiratory disease and abortion, with world-wide distribution. The primary objective of the present study was to describe aspects of BHV-1 infection and control on Irish farms, including herd-level seroprevalence (based on pooled sera) and vaccine usage.

**Methods:**

The characteristics of a diagnostic indirect BHV-1 antibody ELISA test when used on serum pools were evaluated using laboratory replicates for use in the seroprevalence study. The output from this indirect ELISA was expressed as a percentage positivity (PP) value. A proposed cut off (PCO) PP was applied in a cross-sectional study of a stratified random sample of 1,175 Irish dairy and beef cattle herds in 2009, using serum pools, to estimate herd seroprevalence. The study was observational, based primarily on the analysis of existing samples, and only aggregated results were reported. For these reasons, ethical approval was not required. Bulk milk samples from a subset of 111 dairy herds were analysed using the same ELISA. Information regarding vaccine usage was determined in a telephone survey.

**Results:**

A PCO PP of 7.88% was determined to give 97.1% sensitivity and 100% specificity relative to the use of the ELISA on individual sera giving maximization of the prevalence independent Youden's index, on receiver operating characteristics analysis of replicate results. The herd-level BHV-1 seroprevalence was 74.9% (95% CI - 69.9%-79.8%), with no significant difference between dairy and beef herds. 95.5% agreement in herd classification was found between bulk milk and serum pools. Only 1.8 percent of farmers used BHV-1 marker vaccine, 80% of which was live while 75% of vaccinated herds were dairy.

A significant association was found between herd size (quartiles) and seroprevalence (quartiles).

**Conclusions:**

The results from this study indicate BHV-1 infection is endemic, although BHV-1 vaccines are rarely used, in the cattle population in Ireland.

## Background

Infection with bovine herpesvirus-1 (BHV-1) causes a wide range of disease manifestations including respiratory disease, abortion and other less common syndromes [1]. Pathogenicity can vary from mild to severe and relative importance of each syndrome varies between countries. It has a world-wide distribution, though some European countries have a long history of BHV-1 control [2]. A number of Member States within the European Union (EU) have either successfully eradicated BHV-1 (Denmark, Finland, Sweden, Austria, the Italian province of Bolzano-Bozen, Switzerland) or implemented an EU-approved compulsory programme (Germany, the Italian province of Trento). Herd-level antibody prevalence of BHV-1 infection shows a wide variation between countries. The control and eradication of BHV-1 infections has been previously reviewed [3].

In Ireland, some information has recently emerged regarding BHV-1 infection, albeit from a biased subset of Irish beef herds [4] of which 73.2% were seropositive. As yet, dairy herd-level prevalence has not been evaluated, and data are not available concerning strategies used to control infection in Ireland, including vaccination. An understanding of BHV-1 prevalence and vaccine use are necessary for designing and implementing effective national control measures.

The primary objective of this study was to describe aspects of BHV-1 infection and control on Irish farms, including herd-level seroprevalence (based on pooled sera) and vaccine usage. Preliminary validation of an indirect BHV-1 antibody ELISA (SVANOVA; Biotech AB, Uppsala, Sweden) using pooled sera was conducted as part of this study.

## Methods

### Data collection

#### Preliminary validation

Five hundred negative and 500 positive sera ('the archived sera') were selected from routine submissions to the diagnostic unit of Agri-Food and Biosciences Institute (AFBI) in Belfast. The archived sera were assayed using the above mentioned BHV-1 antibody indirect ELISA. These sera were assigned to either of two groups - known positives or negatives. EU standard reference sera (EU-1, EU-2 and EU-3) were used to validate the ELISA for use on single serum samples prior to use in the study [5,6]. The test was performed according to the instructions of the manufacturer. Both positive and negative control sera were included in each assay. Sensitivity (Se) and specificity (Sp) of the test when used on individual sera relative to serum neutralisation test (SNT) are 97.4% and 92.4%, respectively (SVANOVA, data on file).

The archived sera were used to form a series of 'validation pools', each containing 30 sera (20 μL each serum, 600 μL for each sample pool). Specifically, each validation pool included a defined number ('*n*') of positive sera (where *n *= 0, 1, 2, 3, 4, 5, 6, 7, 8, 9, 10, 15, 20, 25 or 30) in combination with 30-*n *negative samples. For example, one validation pool had 0 positive and 30 negative samples, another had 1 positive and 29 negative samples, etc. In total, 90 validation pools were created, including 20 pools where n = 0, and 5 each for the 14 remaining positive/negative combinations. For each of these validation pools, the positive and negative samples were selected using simple random sampling from the archived sera. The 90 validation pools were analysed using the above-mentioned BHV-1 antibody ELISA, all in the diagnostic unit of AFBI. The absorbance or optical density (OD) of each well at 450 nm was measured on a microplate plate reader. The corrected OD (COD) value of each pool and reference serum was obtained by subtraction of the OD value of each control antigen-coated well from that of the parallel viral antigen-coated well [7]. A corresponding percentage positivity (PP) value was obtained using the formula:

PP value = COD (Sample)/COD (Positive Control). Randomisation in this study was performed using a computer-generated random number list (Microsoft Excel 2003).

#### Seroprevalence study

##### Pooled serum

As part of the national statutory brucellosis eradication scheme, serum samples are collected annually from all eligible animals (female bovines and entire bulls aged 12 months or over) in all cattle herds in Ireland. A sample of these herds was selected for the current study. Based on data available through the national Animal Health Computer System database, stratified random sampling (based on two strata: 'province' and 'herd type') was used. There are four provinces in Ireland (Connaught, Leinster, Munster and Ulster). Two herd types were defined in this study - beef (containing > 66% beef breed cows) and dairy (containing > 66% dairy breed cows). The number of animals sampled was proportional to the number of herds with at least one birth registered in 2008 within each stratum. In 2008, 87,396 herds had one or more births registered. Of these, 2,037 were mixed dairy and beef herds and were excluded. A further 30,894 herds were excluded because they were small (dairy herds < 20 breeding cattle; beef < 10 breeding cattle). The proportion of the remaining 54,465 herds within each strata is shown in Table 1. The aim was to select a total sample size of 2688 to allow for random selection of sufficient herds stratified on herd type (beef or dairy) and location (province) (Table 2). This sample size was based on herd-level Se and Sp (calculated from pooled-test Se and Sp values determined in the validation study) [8], a herd-level prevalence based on a previous study of 73% [4], and a participation rate of 50% for farms enrolled in the study during a 10 week collection period. Throughout the study period, animals were mostly kept on managed grassland. While 2688 were initially contacted, 296 herds had to be excluded as they had either already completed their herd test prior to the proposed collection period or their contact details were inaccurate. Hence, 2392 herds were recruited of which 1659 were classified beef (containing < 30% dairy breed cows) and 733 were dairy (> 70% dairy breed cows), with approximately 204,000 animals. These farms represented just over 2% of all beef and dairy herds in Ireland. This represented exclusion of less than 1% of dairy cows, but almost 15% of beef cows [9]. Permission for inclusion in the study was sought from all selected herd owners.

**Table 1:** Proportion of herds with at least one birth during 2008, excluding mixed and small (dairy < 20 animals, beef < 10 animals) herds, by province and herd type

Province	Animals			Herds			% of herds		
	
	Beef	Dairy	Total	Beef	Dairy	Total	Beef	Dairy	Total
Connaught	264,141	51,439	315,580	12,316	1032	13,348	22.6	1.9	24.5

Leinster	300,933	254,292	555,225	9825	3908	13,733	18.0	7.2	25.2

Munster	304,081	621,957	926,038	11,077	10,174	21,251	20.3	18.7	39.0

Ulster	106,809	73,675	180,484	4752	1381	6133	8.7	2.5	11.3

Total	975,964	1,001,363	1,977,327	37,970	16,495	54,465	69.7	30.3	100.0

**Table 2:** Number of beef and dairy herds initially recruited within each province

Province	Herd distribution		
	
	Beef	Dairy	Total
Connaught	608	51	659

Leinster	485	193	678

Munster	547	502	1049

Ulster	234	68	302

Total	1874	814	2688

Sample collection was conducted by recruitment of 382 private veterinary practitioners (PVPs) during a ten-week period from May to August 2009. The study was observational, based primarily on the analysis of existing samples, and only aggregated results were reported. For these reasons, ethical approval was not required. The serum were placed in deep well blocks and stored frozen. Samples were later thawed, and a serum pool was generated for each herd, derived from up to 30 individual sera (each 10 μL). In herds containing < 30 eligible animals, the pool included sera from all animals; in herds containing > 30 eligible animals, 30 sera were randomly selected based on a randomisation list generated in Excel 2003. Sample pooling was carried out in Enfer Diagnostics, Naas, Co. Kildare, Ireland, an officially accredited laboratory. Testing of the serum pools was conducted at AFBI using the same indirect ELISA.

##### Bulk milk

120 dairy herds were selected using convenience sampling for bulk milk analysis. These were the first herds from which serum samples were collected. In total, bulk milk was collected from approximately 20% of all dairy herds from which sera samples were obtained. Using a prepared protocol, each herd-owner collected the bulk milk sample into a 20 mL universal container from an agitated bulk tank. Containers contained bronopol preservative tablets and were posted to AFBI for analysis using the above-mentioned test, for the purposes of comparison with results from the sample serum pools.

#### Vaccine usage survey

A phone survey was used to clarify BHV-1 vaccine usage in each of the study herds. Each study herd keeper was contacted by phone by the first author, and an interview was conducted regarding duration and timing of vaccination and brand of vaccine used. National usage data were obtained from sales data gathered by an industry survey organization [10]. GfK Kynetec is an international market research company that gathers data on sales of animal health products. Using Microsoft Excel 2003, the data were summarised according to the number of vaccine doses sold by time of application (month), and by location (county; 26 in 4 provinces within the Republic of Ireland).

### Data analysis

#### Preliminary validation

A validation pool was classified positive if it contained ≥ 1 positive archived serum sample. The PP values from each of the 90 validation pools was recorded in a statistical software package (STATA^®^, Version 11.0/SE; Stata Corporation, Texas, USA, 2009) and subjected to Receiver Operator Characteristics (ROC) analysis, to determine the optimal cut-off PP to maximize Se and Sp of the test when used on pools. However, only values for Se and Sp when the test is used on individual serum samples are available (and not pool Se (PSe) and pool Sp (PSp)) (Manufacturer data on file). We assume that PSe = Se and PSp = Sp. [11]. Youden's index was calculated using the formula (Se+Sp-1) [12].

#### Seroprevalence study

##### Pooled serum

The OD result of each sample pool was recorded. The optimal cut-off PP value, as determined during preliminary validation, was used to classify each herd as seropositive or negative. Differences in observed prevalence by herd type, herd size (quartiles) and province were tested using a chi-square test.

##### Bulk milk

Bulk milk samples with PP ≥ 3 were deemed positive, as per manufacturer recommendations. The statistical software package: STATA^®^, Version 11.0/SE (Stata Corporation, Texas, USA, 2009) was used to calculate Cohen's kappa coefficient, which is a measure of agreement between the bulk milk results and the seroprevalence results. Herds that had a different result in the bulk milk and seroprevalence studies were further investigated by whole herd analysis of individual serum samples to determine which classification was correct.

#### Vaccine usage

Vaccine usage by seasonality, herd type and brand were evaluated. Vaccine usage in study herds was compared to national usage data.

## Results

### Preliminary validation

**Figure 1:**
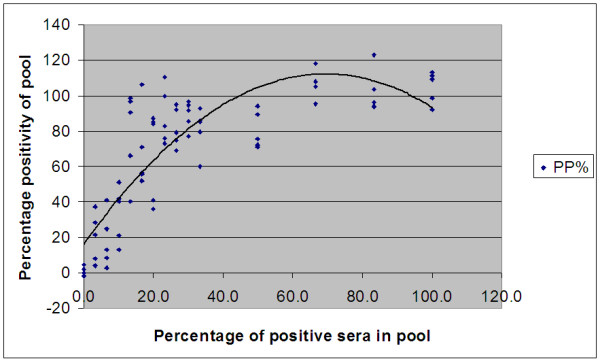
**Comparison between percentage positivity and percentage of positive samples with 90 validation pools**.

**Figure 2:**
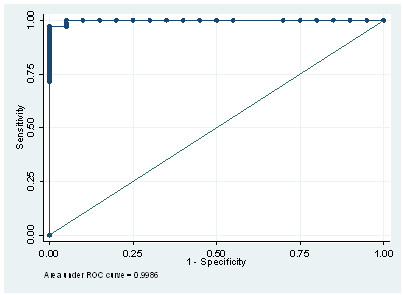
**Receiver operating characteristics curve using percentage positivity measurements of serum pools containing assigned proportions of known positive serum samples**.

There was good agreement between seropositive pools and seropositivity (R^2 ^= 0.70; Figure 1), and a significant association between seropositivity and PP (*P *< 0.001). Based on a ROC analysis of PP readings (Figure 2), a cut-off PP of 2.63 resulted in a Se and Sp of 100% and 95%, respectively, relative to the use of the ELISA on individual sera (Table 3). A proposed cut-off (PCO) PP of 7.88 gave a relative Se and Sp of 97.1% and 100%, respectively. The PCO value was chosen based on maximization of the prevalence independent criterion (Youden's index) in order for the ELISA to be used on bulk serum pools in an Irish seroprevalence study.

**Table 3:** Excerpt of detailed report of sensitivity and specificity from receiver operator characteristic analysis of ELISA percentage positivity of serum pools by % of seropositive samples in pool

Cut-point	Sensitivity (%)	Specificity (%)	Classified (%)	LR+	LR-
(> = -0.2159)	100.00	0.00	77.78	1	

(> = -0.1460)	100.00	5.00	78.89	1.0526	0

(> = -0.1016)	100.00	10.00	80.00	1.1111	0

(> = -0.0889)	100.00	15.00	81.11	1.1765	0

(> = -0.0190)	100.00	20.00	82.22	1.25	0

(> = -0.063)	100.00	25.00	83.33	1.3333	0

(> = 0.0)	100.00	30.00	84.44	1.4286	0

(> = 0.0127)	100.00	45.00	87.78	1.8182	0

(> = 0.0190)	100.00	50.00	88.89	2	0

(> = 0.0381)	100.00	55.00	90.00	2.2222	0

(> = 0.0508)	100.00	60.00	91.11	2.5	0

(> = 0.0762)	100.00	65.00	92.22	2.8571	0

(> = 0.0889)	100.00	70.00	93.33	3.3333	0

(> = 0.1714)	100.00	75.00	94.44	4	0

(> = 0.1840)	100.00	80.00	95.56	5	0

(> = 0.1841)	100.00	85.00	96.67	6.6667	0

(> = 0.2413)	100.00	90.00	97.78	10	0

(> = 0.2628)	100.00	95.00	98.89	20	0

(> = 0.4008)	98.57	95.00	97.78	19.714	0.015

(> = 0.4402)	97.14	95.00	96.67	19.429	0.0301

(> = 0.7884)	97.14	100.00	97.78		0.0286

(> = 0.8344)	95.71	100.00	96.67		0.0429

(> = 1.2746)	94.29	100.00	95.56		0.0571

(> = 1.3009)	92.86	100.00	94.44		0.0714

(> = 2.0959)	91.43	100.00	93.33		0.0857

(> = 2.1419)	90.00	100.00	92.22		0.1

### Seroprevalence study

#### Pooled serum

Sera from 1,175 herds were collected by 199 PVPs. Participation was largely influenced by the decision of the herd-owner on the most suitable seasonal timing of their brucellosis herd test, with 1217 herds choosing to delay until after the collection period, mainly for commercial reasons. Approximately 61,000 sera (from about 60% of all bovine animals in these herds) were collected during the study period. Of the collected herds, 450 contained 30 or less samples. Using the PCO determined in the validation study, 295 herds were classified as seronegative. The highest PP readings in each quartile were 7.3, 67.2, 95.9 and 159% respectively. The herd-level BHV-1 prevalence was 74.9% (95% CI - 69.9%-79.8%). Herd level prevalences by province and herd type are summarized in Table 4. There was a significant difference between provinces for herd-level BHV-1 seroprevalence (*P *< 0.001), with highest seroprevalence in Leinster and lowest in Munster. The seroprevalence for dairy and beef herds was not significantly different (*P *= 0.868: 74.6% vs 75.0%). Herd-level BHV-1 seroprevalence was significantly associated with herd size (Table 5).

**Table 4:** Herd level prevalence within each province, by herd type

	Prevalence - Number of seropositive herds (%)		
**Province**	**Beef**	**Dairy**	**Total**

Connaught	169 (70.9)	18 (85.7)	187 (72.1)

Leinster	176 (84.5)	87 (83.7)	263 (84.2)

Munster	173 (70.2)	165 (69.2)	338 (69.7)

Ulster	68 (77.3)	25 (78.1)	93 (77.5)

Total	586 (75.0)	295 (74.6)	881 (74.9)

**Table 5:** Chi-squared testing of herd-level bovine herpesvirus-1 (BHV-1) seroprevalence (into quartiles) by herd size (in quartiles), in 1052 Irish study herds during 2009

Herd-level BHV-1 seroprevalence	Herd size				Total
		
	Lowest quartile	Second quartile	Third quartile	Highest quartile	
Lowest quartile	98^a^	58	52	54	262

Second quartile	72	85	64	46	267

Third quartile	67	61	75	60	263

Highest quartile	55	43	64	98	260

Pearson Chi-square (9) = 57.1295, *P *< 0.001

a. That is, there were 98 herds with herd-level BHV-1 seroprevalence in the lowest quartile and herd size in the lowest quartile

#### Bulk milk

In total, 111 bulk milk samples were collected. The highest PP readings in each quartile were 1.2, 37.7, 97.1 and 153% respectively. A comparison of the results from the pooled serum and bulk milk analyses, using the two different cut-offs, is presented in Table 6. Subsequently, the more-conservative PP of 7.88 was used to determine herd-based BHV-1 seroprevalence. Kappa analysis demonstrates 95.5% agreement between herd classification of seroprevalence based on pooled serum (73.8%) and bulk milk (71.2%) analysis (Table 6) when PCO is applied. Misclassification occurred with 5 herds (4 were pooled serum +ve, bulk milk -ve; 1 the converse). In further evaluation conducted in 2 herds based on an analysis of whole herd individual serum, one had been misclassified as negative using bulk milk analysis (that is, one positive animal in a herd of forty one) and the other misclassified as negative using pooled serum analysis (six positive animals in a herd of 124 animals). While the latter observation is in line with previous findings [13], it must be clarified that the bulk serum pool contained only two of the positive animal samples.

**Table 6:** Comparison of bulk milk and pooled serum bovine herpesvirus-1 (BHV-1) results from 111 herds in Ireland during 2009

BHV-1 herd status based on bulk milk analysis (PP ≥ 0.03)	BHV-1 herd status based on pooled serum analysis			
	
	(PP 2.63)		(PP 7.88)	
	
	Negative	Positive	Negative	Positive
Negative	26	6	28	4

Positive	1	78	1	78

κ-value	0.839		0.887	

The pooled serum results are separately presented at two different cut-off percentage positivity (PP) values (2.63 and 7.88)

### Vaccine usage

**Figure 3:**
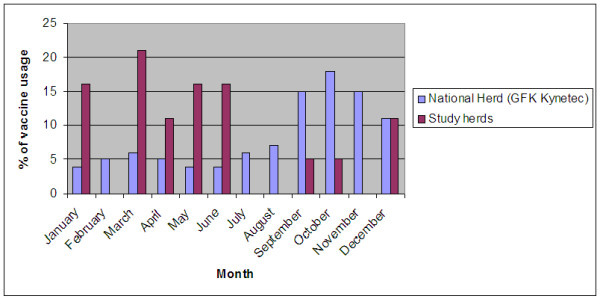
**Seasonality of bovine herpesvirus-1 vaccine usage in Ireland during 2009, both nationally and in the study herds**.

Interviews were conducted with 1,113 (94.7% of the) study herd keepers. Of these, 20 (1.8%) used BHV-1 marker vaccine (80% live and 20% inactivated). 75% of vaccinated herds were dairy. In total, 50% and 25% of the vaccinated herds had been vaccinating for < 1 and > 1 but < 2 years, respectively. Approximately 470,000 doses of BHV-1 marker vaccine were used in the Irish market in 2009 (85% live) [10], with a distinctly seasonal pattern of usage (60% of usage during the months August to December) [10]. The seasonality of vaccine usage is summarized in Figure 3.

## Discussion and Conclusions

Apparent herd-level seroprevalence of BHV-1 in Ireland was 74.9%, which is in agreement with previous findings for bulls [4]. Assuming a test Se and Sp of 97.4% and 92.4%, respectively, the true herd-level prevalence is 77.4% (74.98-79.76 - 95% CI). This obviously compares unfavourably to levels in EU member states that are officially free while levels in other member states where information is available are broadly similar to Ireland [3]. Though not specifically investigated in this study, it is likely that such high levels in Ireland result from historic management practices and particularly the dispersal of animals throughout the country in the context of national trading at markets [14]. However, this issue warrants further investigation. Herd-level BHV-1 seroprevalence was significantly associated with herd size which is in line with previous findings, though this may be a cluster risk factor [15,16].

It is apparent from study herds that vaccine usage in breeding animals in Ireland is very low (1.8%). Regarding control measures implemented at herd level, only vaccination was investigated in this study. Recall bias could have arisen among farmers surveyed. Vaccine brand used was determined to minimize recall bias and confusion with vaccines used to control other diseases. Furthermore, a major difference exists between vaccine usage in the national cattle population and study herds. Approximately 471,000 doses of BHV-1 vaccine were used in the Irish market in 2009 [10]. It is estimated that the majority of vaccine is used in growing animals (< 2 years old) prior to housing/shipment rather than breeding animals. This would explain the distinctly seasonal usage of BHV-1 marker vaccine in the Irish cattle population prior to housing or export of animals in the months September to November. This pattern is not followed in study herds, as included herds consist of breeding animals. The low number of study herds vaccinating precluded investigating any link between seroprevalence and vaccine uptake.

True herd-level seroprevalence of BHV-1 can be determined from the number of seropositive pools [8]. The effects of pooling on Se and Sp of tests have been evaluated previously [8,17]. When a single pooled sample is collected per herd, the only question that can be answered is whether the herd is infected or not. Sampling bias from PVPs refusing to participate was minimised by encouraging participation through clear communication of objectives to participating PVPs with a commitment to provide herd-level results. Procedures were implemented to avoid cross-contamination and to ensure minimization of the effect of cross-contamination. Cross-reaction with other agents is possible if a pool contained a viraemic animal. The prevalence of viraemic animals in this study is unknown. However, it has been suggested that pooling can reduce the impact of cross-reaction due to the dilution of cross-reacting agents with samples from non-infected animals [8]. Few publications exist on the use of ELISA tests on serum pools [18,19], though pools of 10 are widely used by the Agence Française de Sécurité Sanitaire des Aliments in France to assess herd-level prevalence of disease [20]. It has been found in a previous study that 16.3% of seropositive herds had only one cow seropositive while an additional 8.6% had only two cows seropositive [21]. This means that there is a significant risk that herd-level prevalence will be under-estimated, using serum pools containing only 10 animals. Therefore, up to 30 animals in each herd were used in the serum pools in this study. Furthermore, bulk milk sample analysis agreed significantly with findings of the same herd bulk serum test (Table 6). While desirable, analysis of all samples individually would have been prohibitively expensive.

The preliminary validation study was carried out to provide a PCO for use on serum pools of 30 samples. It is reasonable to assume that this cut-off is applicable to other serum pools of equal or lower size and could provide a useful cost-reducing tool in prevalence studies using this ELISA. Two such cut-offs were found on ROC analysis of the results and the higher cut-off (PCO) was used, to maximize the Youden's index, as a conservative approach to determining national herd-level seroprevalence. Furthermore, PCO gave better agreement when compared to results obtained in the bulk milk study (κ = 0.887 compared to 0.839).

As herd classification is based on pooled tests in the seroprevalence study, estimation of herd-level Se and herd-level Sp is more complex because assumptions must be made about PSe and PSp. In this study it was assumed PSe = Se and PSp = Sp. However, it is likely that the PSe would be lower than Se especially when within-herd prevalence is low and pool size is large. The dilution effect on PSe will also be dependent on the exposed animal's concentration of antibody. In this study, one positive serum sample in the pool (equivalent to 3% within-pool prevalence) was used to determine cut-off in the ROC analysis. Furthermore, within-herd prevalence in the seroprevalence study is likely to be approximately 28% [4] Individual animals remain seropositive for a relatively long time after infection with maintained high levels of antibodies [22]. It is therefore contended that any effect of pool size on PSe will be mitigated by a combination of these factors. Conversely, PSp should exceed Sp because dilution should make it less likely to have a false-positive pooled test result than a false-positive individual-test result [8]. Finally, there is 95.5% agreement in herd classification between bulk milk and serum pools, using a manufacturer-recommended and validated cut-off PP for bulk milk [23]. However, larger-scale work is advised on the use of serum pools with low seroprevalence to confirm the cut-off found in this study and to more accurately validate the use of this test on serum pools. Finally, it is important to emphasise that the validation of the ELISA test on serum pools was performed against the same ELISA used on individual samples. While this is not optimal, as the SNT is the accepted gold standard test for validation, large quantities of serum with known SNT readings were not available. Many laboratories no longer routinely carry out this test as it is time and labour-intensive and requires specialist skills and equipment for interpretation. However, the ELISA has been validated by the manufacturers against the SNT when used on individual sera, with a Se and Sp of 97.4% and 92.4%, respectively.

Significant future challenges exist in Ireland regarding BHV-1 infection with herd size increasing and no national control programme in place [4]. Two production strata for cow farms are described in Ireland - beef suckler farms (n = 74,800) containing 1.105 million cows and dairy farms (n = 26,800) containing 1.087 million dairy cows, with average herd size 15 and 41, respectively [24]. A small proportion of herds are both dairy and beef suckler [25]. Numbers of farms are falling by 3-4% per year as average farm size increases. The low uptake of vaccine in herds of adult cows will do little to restrict virus circulation among adult or juvenile stock. Vaccination has been demonstrated to effectively reduce seroprevalence if vaccine usage is conducted in a coordinated manner [26]. In addition to those EU member states already officially free of disease, other countries have already achieved regional eradication (France, Germany, UK, Spain and Italy) or are at various stages of herd certification/eradication (Netherlands, Belgium and Poland among others) [27]. Further efforts are required by Animal Health Ireland and other agencies in the Republic of Ireland in order to avoid high herd-level prevalence of BHV-1 acting as a potential barrier to within-community trade [28]. These would include increasing awareness of this prevalent disease and encouraging implementation of cost-effective controls, including screening, vaccination (particularly of breeding animals), biosecure practices and a herd accreditation programme.

## Abbreviations

AFBI: Agri-Food and Biosciences Institute, Stormont, Belfast, Northern Ireland; ANOVA: Analysis of variance; BHV-1: Bovine Herpesvirus-1; CI: Confidence Interval; COD: Corrected Optical Density: The value of each sample and reference sample was obtained by subtraction of the OD value of each control antigen-coated well from that of the parallel viral antigen-coated well in the ELISA; ELISA: Enzyme-linked immunosorbent assay; EU: European Union; κ: Cohen's kappa coefficient; μL: Microlitre; OD: Optical Density: The absorbance of each sample well at 450 nm was measured on a microplate plate reader; PCO: Proposed cut-off; PP: Percentage Positivity; PVPs: Private Veterinary Practitioners; ROC: Receiver Operator Characteristics analysis,; Se: Sensitivity; Sp: Specificity; SNT: Serum Neutralisation test

## Competing interests

The corresponding author is an employee of MSD Animal Health (Ireland), however, the company played no role in study design, data collection and analysis, decision to publish, or preparation of the manuscript. The remaining author(s) declare that they have no competing interests.

## Authors' contributions

DJBC conceived and designed the study, organised the blood sample collection and analysis, designed and conducted the owner questionnaires, collected vaccine usage data and drafted the manuscript. SJM and TAC participated in the design and coordination of the study, and DJBC and TAC performed the statistical analysis. SJM and MLD helped to draft the manuscript. All authors read and approved the final manuscript.

## Authors' information

DJBC is a Bachelor of Veterinary Medicine and works as Technical Manager, MSD Animal Health (Ireland). TAC works as principal statistician in the Centre of Veterinary Epidemiology and Risk Analysis (CVERA), within the School of Agriculture, Food Science and Veterinary Medicine at University College Dublin (UCD). MLD is UCD Professor of Veterinary Clinical Studies. SJM is UCD Professor of Veterinary Epidemiology and Risk Analysis and CVERA Director.
